# Advances in the management of osteosarcoma

**DOI:** 10.12688/f1000research.9465.1

**Published:** 2016-11-25

**Authors:** Stefan S. Bielack, Stefanie Hecker-Nolting, Claudia Blattmann, Leo Kager

**Affiliations:** 1 Klinikum Stuttgart - Olgahospital, Zentrum für Kinder-, Jugend- und Frauenmedizin; Pädiatrie 5 (Onkologie, Hämatologie, Immunologie), Kriegsbergstrasse 62, Stuttgart Cancer Center, Germany; 2 St. Anna Children's Hospital, Department of Paediatrics, Medical University Vienna and Children's Cancer Research Institute CCRI, Vienna, Austria

**Keywords:** osteosarcoma, imaging, surgery, chemotherapy, radiotherapy

## Abstract

Osteosarcoma, a bone cancer most commonly seen in adolescents and young adults, is usually a high-grade malignancy characterized by a very high risk for the development of pulmonary metastases. High-grade osteosarcomas are usually treated by preoperative and postoperative chemotherapy and surgery, with a very limited number of active agents available. Rarer lower-grade variants such as parosteal and periosteal osteosarcoma or low-grade central osteosarcoma are treated by surgery only. Imaging to search for possible metastases focuses on the lung. Computed tomography is the most sensitive method but cannot reliably distinguish small metastases from benign lesions. Advances of local imaging and surgical reconstruction now allow the use of limb-salvage in an ever-increasing proportion of patients. While still troubled by complications, non-invasive endoprosthesis-lengthening mechanisms have led to an increased uptake of limb-salvage, even for young, skeletally immature patients. Radiotherapy is employed when osteosarcomas cannot be removed with clear margins, but very high doses are required, and both proton and carbon-ion radiotherapy are under investigation. Unfortunately, the past 30 years have witnessed few, if any, survival improvements. Novel agents have not led to universally accepted changes of treatment standards. In patients with operable high-grade osteosarcomas, the extent of histological response to preoperative chemotherapy is a significant predictive factor for both local and systemic control. Attempts to improve prognosis by adapting postoperative treatment to response, recently tested in a randomized, prospective setting by the European and American Osteosarcoma Study Group, have not been proven to be beneficial. Many agree that only increased knowledge about osteosarcoma biology will lead to novel, effective treatment approaches and will be able to move the field forward.

## Introduction

A 1.6–1.8-million-year-old hominin metatarsal from the South African Swartkrans paleoanthropological cave site makes osteosarcoma the earliest documented cancer of humankind ^1^. Unfortunately, the only time period since then during which significant prognostic gains were achieved was from the late 1970s until the early 1980s, when combining multi-agent chemotherapy with surgery revolutionized treatment ^2^. Unfortunately, the decades since have witnessed no further improvements of survival in North America ^3^ or Europe ^4– 6^. Nevertheless, there have been numerous advances in the management of osteosarcoma which merit review and discussion.

Osteosarcoma is a rare bone cancer which mainly affects adolescents and young adults. Though lower-grade variants exist, most are high-grade malignancies with a high propensity for lung metastases. Current standard treatment consisting of surgery plus chemotherapy leads to long-term, disease-free survival in approximately 60% of patients with localized extremity disease ^7– 10^ and 20–30% for patients with primary metastases or axial primaries ^7, 11^. Most patients are treated using a neoadjuvant approach, and histologic response to preoperative chemotherapy has emerged as an independent prognostic indicator ^7^. While combined preoperative and postoperative chemotherapy has never been shown to provide survival benefits over adjuvant chemotherapy alone (as long as both contain the same cumulative doses) ^7, 12^, it offers time to prepare for surgery and allows an *in vivo* evaluation of the effects of systemic treatment. These may be estimated by a variety of imaging methods, but histologic assessment for the proportion of viable tumor remaining at surgery is the gold standard. Patients whose primaries respond well to chemotherapy, usually defined as <10% tumor viability, generally suffer fewer local ^13^ and systemic ^7, 9, 10^ recurrences and achieve greater survival probabilities ^7^ than others. This manuscript will try to highlight recent advances achieved in this context of first-line treatment.

## Advances in imaging

Imaging of bone sarcomas was revolutionized by magnetic resonance imaging (MRI), which, for the first time, allowed detailed assessment of tumor extent within the bone marrow cavity and into soft tissues, as well as its relation to surrounding structures such as joints, nerves, and vessels. In addition, MRI may also be used to predict histologic tumor response to preoperative chemotherapy, as may positron emission tomography (PET)/computed tomography (CT), sequential bone scans, and others. PET/MRI has entered the scene more recently ^14^, and its role remains to be defined. The importance of accurate imaging at initial diagnosis and after preoperative chemotherapy, however, cannot be overstated. A detailed review of local imaging would be beyond the scope of this article and the reader is referred to the recent literature ^15– 19^.

Chest CT remains the gold standard for imaging lung metastases ^20^. Unfortunately, even modern CT scanning cannot reliably discriminate small lung metastases from small benign lesions. A recent study of 283 CT-identified lesions which led to 123 thoracotomies in 70 osteosarcoma patients found 234 of the lesions to be metastases. An additional 31, 14 of those metastases, were identified only upon thoracotomy. Lesion size ≥6 mm was suggestive for metastases, but many smaller lesions were also malignant. While most metastases were nodular and calcified, atypical findings were common ^21^. This analysis again confirms that chest CT has its limitations. Given the dire consequences associated with incomplete resection of metastatic osteosarcoma ^11^, we believe that any lung lesion detected by CT should be viewed with a high index of suspicion and treated as if it could be a metastasis. However, as highlighted by unrelated surveys among leading study groups ^22^ and members of the Connective Tissue Oncology Society ^23^, the jury is still out and considerable variability surrounds the management of pulmonary lesions.

A minority of osteosarcomas will present with synchronous bone metastases; ^99m^Technetium bone scans have long been part of the standard diagnostic workup. Some years ago, whole-body MRI with short time inversion recovery (STIR) imaging was found to be more sensitive for detecting bone metastases in children with suspected multifocal bone lesions than bone scans, but also less specific ^24^. Similar observations were made for PET/CT: in a recent series of 39 osteosarcomas investigated by 40 paired bone scans and PET/CTs and of whom five had bone metastases, PET/CT detected all, while bone scans missed two. On the other hand, three PET/CTs were falsely positive ^25^. It seems that histologic confirmation with a biopsy is often required before an osseous lesion suspected by whole-body STIR–MRI or PET/CT is considered a true bone metastasis but that bone scans will usually not detect additional lesions in patients investigated by either of those techniques.

## Advances in biopsy techniques

Osteosarcoma must be confirmed histopathologically before initiating tumor-directed therapy. Biopsies were traditionally performed via incisional procedures. Even though scientists may lament a paucity of tissue for research, less invasive core needle biopsies (CNBs) are now assuming an ever-increasing role. These have been shown to be very effective as long as adequate cores can be sampled. A French analysis of CNB in 73 osteosarcomas reported an overall sensitivity of 93.1%, specificity of 100%, and positive and negative predictive values of 100% and 99.9%, respectively, as long as the specimen was adequate ^26^. CNB does not seem to be nearly as reliable in telangiectatic osteosarcoma: in one series, nine of 26 were misdiagnosed as aneurysmal bone cysts ^27^.

## Advances in local therapy of operable osteosarcoma

Surgery with wide margins ^28^ remains the mainstay of curative local therapy. Spurred by major advances of imaging and of surgical reconstruction opportunities, recent years have witnessed a major shift from amputations towards limb-saving procedures ^29^. Limb-salvage, however, poses challenges, particularly in growing individuals. Earlier models of expandable endoprostheses required additional surgery for every lengthening. Various non-invasive lengthening mechanisms are now available, including incorporated engines or magnetic devices ^30^. However, these are still associated with frequent complications and needs for revisions ^31– 33^. Several papers emphasize that further technical advances are direly needed: in one series, 10 patients experienced 37 implant-related complications ^31^, and in another, 42% of 38 patients experienced complications, including 10 prosthesis revisions and two amputations ^34^. A third reported an average 2.5 revisions for complications in 71 patients ^32^; a fourth even questioned whether complications associated with a particular, rather popular, expandable prosthesis were acceptable for its continued use ^33^. Given that such devices are obviously still immature, one might look back to the bygone age of rotationplasties with (never expected) nostalgia. Their function remains quite good even with long observation periods. A recent Italian series evaluated 25 patients living with rotationplasties for a mean of 15 years ^35^. While arthritis of the tibiotalar, subtalar, and talonavicular joints was radiographically present in most, they showed improved gait parameters as adults compared with previously reported findings for children with rotationplasty ^35^.

## Advances in local treatment of inoperable osteosarcomas

Any osteosarcoma that can be operated on should be operated on to maximize the chance for local control and hence survival. However, not all osteosarcomas are operable. Several series have confirmed that selected patients may achieve permanent local control with radiotherapy, particularly if this is combined with effective chemotherapy and gross total resection ^36, 37^. Results of a meta-analysis suggest that debulking may no longer be required when radiation doses of 70 Gy or higher are administered. Local control probabilities after radiotherapy were lower for craniofacial osteosarcomas than for those of other sites ^37^.

The high radiation doses required to sterilize osteosarcoma are difficult to achieve with conventional techniques, so that proton and heavy-ion radiotherapy have come into focus. In probably the largest series of 55 osteosarcomas treated with protons, the mean total radiation dose was 68.4 Gy. At 5 years, the local control rate was 72% and overall survival was 67% ^37^. Among 78 patients with inoperable osteosarcoma of the trunk irradiated with a median of 70.4 Gy carbon-ion radiotherapy (CIRT) by Japanese investigators, the 5-year local control rate was 62% ^38^. Osteosarcomas were also included in an array of sarcomas of the spine ^39^ or extremities ^40^ treated with CIRT. While the observed results were also encouraging, further research is required before such techniques can be considered standard. A systematic review of clinical outcome studies published between 2007 and 2015 concludes that there is insufficient evidence on the long-term effectiveness and harm of protons to either support or refute their use in children with osteosarcoma or basically any other pediatric cancer ^41^.

## Advances in systemic treatment

Systemic therapy for osteosarcoma has changed very little for over 30 years and still relies on varying combinations incorporating several of the same four “old” drugs, namely high-dose methotrexate (HD-MTX), doxorubicin (Adriamycin), cisplatin, and ifosfamide ^9, 10, 15, 42^. The MAP combination of HD-MTX, doxorubicin, and cisplatin ^43– 45^ is frequently used, but similar results have been achieved with other protocols employing several of the mentioned agents ^9, 10^. A meta-analysis of published osteosarcoma trials concluded that using three of the drugs led to better results than using only two but that administering all four did not lead to further improvements ^46^.

Several prospective trials have attempted to introduce additional agents for either all patients or certain risk groups. Some years ago, the prospective randomized INT0133 trial addressed two potential additions to MAP using a randomized two-by-two factorial design: the cytotoxic agent ifosfamide and the macrophage activator liposomal muramyl tripeptide phosphatidylethanolamine (L-MTP-PE, mifamurtide). A first publication ^43^ as well as a second ^44^ concluded that there was no benefit of adding ifosfamide. The two papers differed in their conclusions regarding L-MTP-PE: while the first stated that analysis was prevented by an interaction between ifosfamide and L-MTP-PE ^43^, the authors no longer detected a statistically significant interaction 3 years later and decided to examine each intervention separately, as originally planned. They now reported a non-significant trend toward better event-free survival (EFS) and improved overall survival with L-MTP-PE ^44^. Commentators voiced interaction concerns and questioned whether INT0133’s results met generally accepted standards for practice-changing conclusions. They called for additional clinical evaluations to define the role of the drug and to demonstrate whether any potential benefit requires concurrent use of ifosfamide ^47^. We along with others have also argued for additional randomized comparative evaluation to substantiate the utility of the drug ^48^. Since then, however, little new evidence concerning its potential efficacy has emerged. Results from the metastatic cohort of INT0133 pointed in the same direction as in non-metastatic patients but were not statistically significant ^49^. Come 2016, there is additional evidence that L-MTP-PE has a favorable safety profile: a patient access study of 200 patients reported 3,679 infusion-related adverse events after 7,482 infusions, commonly chills, fever, headache, and fatigue, but only rarely severe ^50^. However, there have been no further trials which shed more light upon the potential efficacy of the drug, so uncertainties remain regarding its potential role.

Another drug with immunological properties (along with many other potential mechanisms of action ^51^), interferon alpha-2b, was investigated in the largest prospectively randomized osteosarcoma study to date, the European and American Osteosarcoma Study Group (EURAMOS)-1 trial ^45, 52, 53^. A total of 716 patients whose resectable localized or primary metastatic osteosarcomas responded well to preoperative MAP were randomized after surgery to four additional cycles of MAP either with or without maintenance pegylated interferon alpha-2b ^53^. Of 357 patients randomized to receive the study drug, 271 actually started, of whom 105 stopped early. As expected, for patients whose osteosarcomas responded well to chemotherapy, 3-year EFS for all randomized patients was favorable at 76%. The hazard ratio from an adjusted Cox model was 0.83, but the 95% confidence interval (CI) included 1, meaning that MAP plus interferon alpha-2b was not statistically different from MAP alone. Interpretation of the data is, of course, complicated to a certain extent by the relevant proportion of patients who never started or who prematurely stopped interferon alpha-2b. Nevertheless, the results do not argue for its inclusion in standard osteosarcoma treatment ^53^.

Encouraging results with the combination of high-dose ifosfamide and etoposide were reported from phase II trials of primary or relapsed metastatic osteosarcoma ^54, 55^, so that postoperative addition of the combination to MAP (MAPIE) was investigated in the poor responder cohort of EURAMOS-1 ^45, 52^. In this trial, MAPIE patients were to receive an additional three courses of 14.000 mg/m ^2^ ifosfamide with 500 mg/m ^2^ etoposide and two courses of ifosfamide at 9.000 mg/m ^2^ added to doxorubicin. MAPIE lasted 11 weeks longer than MAP. The study sought to detect absolute improvements of 10% from 45% to 55% in 3-year EFS and 5-year overall survival (hazard ratio 0.75) ^56^. Of 618 randomized patients, 310 were allocated to postoperative MAP and 308 to MAPIE; 3-year EFS rates were 55% (95% CI 49–60) and 53% (95% CI 47–59), respectively. MAPIE was more toxic and fewer patients received their intended chemotherapy doses. MAPIE was also associated with higher risk of secondary malignancy, predominantly leukemia, mostly with cytogenetic abnormalities associated with the administration of alkylating drugs (monosomy-7 or chromosome-5 abnormalities) or etoposide (11q23 abnormalities). Therefore, the EURAMOS consortium argues against adding ifosfamide and etoposide to the MAP backbone of MAP therapy for patients whose osteosarcoma shows a poor response to preoperative treatment ^56^.

Anyone arguing that the alkylator doses used in EURAMOS-1 were not sufficient and that high-dose chemotherapy (HDCT) with autologous blood stem cell transplant (ASCT) was a better idea should be duly cautioned by results from recent uncontrolled prospective trials: in an American study, 18 patients with newly diagnosed localized high-grade osteosarcoma and poor histologic response received HDCT/ASCT with melphalan and cyclophosphamide; 5-year EFS and overall survival were 28% and 48%, respectively ^57^. A Scandinavian–Italian study investigating postoperative high-dose carboplatin/etoposide with ASCT involved 71 patients with primary metastatic or axial osteosarcoma, of whom 29 received two and 10 one course of HDCT; 5-year EFS and overall survival were 27% and 31%, respectively. When patients not receiving HDCT owing to disease progression were excluded, there were no differences in outcomes between patients who received HDCT or not ^58^.

A completely different drug, the bisphosphonate zoledronate, was investigated in the prospective, randomized French multi-center OS2006 trial, which asked whether 10 courses of zoledronate added to chemotherapy and surgery might improve EFS ^59^. Chemotherapy used in this trial varied by age. Among 318 patients, 55 with primary metastases, 160 were randomized to zoledronate. The trial was stopped for futility after the second planned interim analysis when 3-year EFS was 57% for the zoledronate group and 63% for controls (p=0.094) ^59^. While the use of different chemotherapy backbones for different patients might confound interpretation to a certain degree, these results argue against zoledronate's ability to improve oncologic outcomes in osteosarcoma.

OS2006 as well as EURAMOS-1 exemplify that prospective randomized trials are essential to adequately assess whether treatments which show promise in the lab or in early phase studies will truly increase cure rates. They also demonstrate that such trials are feasible, even in very rare cancers such as osteosarcoma.

In summary, there is currently no evidence whatsoever that altering postoperative treatment in patients whose osteosarcomas respond poorly to preoperative chemotherapy or that modifying standard systemic treatment for other reasons will lead to anything but additional side effects and risks. The use of such approaches should be limited to prospective trials and otherwise discouraged.

## Advances in treating osteosarcoma variants

While multi-modal treatment consisting of surgery and chemotherapy is the undisputed treatment standard for patients with high-grade central (arising within the affected bone) osteosarcoma of the extremities or axial skeleton, some osteosarcoma variants deserve special consideration. As for all osteosarcomas, surgery should strive to achieve wide margins. The role of additional systemic treatment, however, varies: low-grade central osteosarcoma arises within bone, just like high-grade central osteosarcoma. These tumors may carry areas of de-differentiation, and the decision to employ chemotherapy is often made based on the most malignant component. An Italian series of 132 low-grade central osteosarcomas included 33 in which high-grade (grade 3) areas were detected in the resected specimen, and postoperative chemotherapy was given to 22 of these 33. High-grade areas accounted for less than 50% in 20/33, among whom only one in nine patients not receiving chemotherapy (unrelated causes) and one in 11 receiving chemotherapy (metastatic recurrence) died. Among 13 of the 33 patients with >50% grade 3 component, 12 received adjuvant chemotherapy, two had local recurrences, and four had metastatic recurrences. The only patient from this cohort treated by surgery only survived disease free ^60^. Similarly, a Norwegian nationwide cohort which included 29 low-grade central osteosarcomas, four of those with areas of de-differentiation, reported 5-year sarcoma-specific survival of 93% and confirmed that low-grade osteosarcoma has an excellent prognosis when resected with a free margin ^61^. These series suggest that low-grade osteosarcomas with small high-grade foci may still be treated by surgery only. The numbers are too small to draw conclusions for low-grade central osteosarcomas which contain larger high-grade areas.

Parosteal osteosarcoma is a low-grade surface osteosarcoma which may also contain high-grade areas ^8^. The already-mentioned Norwegian series also included 20 parosteal osteosarcomas, eight with signs of de-differentiation, and reported 90% 5-year sarcoma-specific survival ^61^. A retrospective British analysis of 80 parosteal osteosarcomas observed overall survival of 92% and 88% at 5 and 10 years, respectively. Local recurrences were associated with intralesional surgery, were de-differentiated in 80%, and were associated with inferior survival. The authors observed neither medullary involvement nor the use of chemotherapy to correlate with survival ^62^. One may conclude that, similar to low-grade central osteosarcoma, adequate surgery is the treatment of choice for parosteal osteosarcoma, that it is imperative to avoid local failure, and that there is no proven role for chemotherapy.

Periosteal osteosarcoma is a surface osteosarcoma of intermediate malignancy ^8^. While sometimes treated with chemotherapy in addition to surgery, the currently available retrospective evidence suggests that treatment should be surgery only. Corroborating similar findings from a 2005 European survey ^63^, an Italian series reported a 10-year overall survival of 84% for 33 patients, 14 of whom received chemotherapy. The authors did not find survival to be influenced by chemotherapy ^64^.

Craniofacial osteosarcomas carry a comparatively high local recurrence risk. The benefit of adding systemic treatments to surgery is not as well defined as for other sites, and no prospective data on adjuvant therapy has recently emerged. Nevertheless, current guidelines suggest using the same multimodal approach as for other high-grade osteosarcomas ^65^.

## Advances in follow-up

Osteosarcoma recurrences may still be cured as long as they are operable ^66, 67^. The aim of tumor-directed follow-up is therefore to detect local recurrences or metastases while surgery is still feasible ^68^. Surveillance usually includes chest X-rays or chest CTs in addition to history, physical, and imaging of the former primary tumor site. The wide variability of surveillance protocols actually employed is exemplified by a Musculoskeletal Tumor Society (MSTS) survey, where the number of first-year surveillance visits ranged from three to six, chest X-rays from zero to three, chest CT scans from one to four, and X-rays of the former primary site from three to six ^69^. Imaging guidelines developed by the Children’s Oncology Group (COG) suggest a schedule which heavily relies on repeated CT scanning of the lungs ^20^. However, conventional chest CT is associated with considerable radiation exposure, which led to criticism of these guidelines ^70^.

Several recent studies have attempted to lend more of an evidence base to osteosarcoma-directed follow-up. A retrospective single-center analysis of 101 patients with routine chest X-ray surveillance reported 34 recurrences. All eight local recurrences were noted clinically, and only two of all recurrences developed beyond 5 years. The authors propose more frequent surveillance visits during the first 2 years and chest X-ray instead of chest CT ^68^. A randomized follow-up study from India investigated 495 patients operated on for seemingly localized primary or recurrent extremity sarcomas (359 of these bone sarcomas). Chest X-ray was compared with CT scanning and 6-monthly with 3-monthly follow-up ^71^. The authors concluded that chest X-rays were not inferior to CT scans in terms of detecting pulmonary metastases and did not lead to inferior survival; 3-year overall survival was 64% with 6-monthly and 69% with 3-monthly follow-up, respectively ^71^.

Our interpretation of the currently available evidence is that routine follow-up for lung metastases can usually be performed with chest X-rays. Ultralow-dose CT, which limits radiation exposure to the equivalent of chest X-rays in two planes, has shown promise for lung cancer screening ^72^, so this may change should further studies demonstrate benefits for this technique in the follow-up of bone sarcoma.

## Future outlook

Members of the generation of doctors who saw osteosarcoma cure rates rise within their professional lifetimes have by now reached retirement or are close. How do we move forward? The optimal “conventional” chemotherapy regimen remains to be defined, and efforts to identify additional effective cytotoxic combinations, as exemplified by the demonstration of activity for the gemcitabine/docetaxel combination ^73^, or to augment the usability of known effective agents by mitigating toxicities, exemplified by adding the cardioprotective agent dexrazoxane to increase doxorubicin exposure ^74^, are ongoing. It would be very optimistic to expect anything but limited improvements from such approaches.

Like in most other cancers, immunotherapy and the so-called targeted therapies are current hot topics ^75– 78^. As exemplified in 29 patients with refractory/relapsed osteosarcomas registered between 2009 and 2013 within the French Sarcoma Group–Bone Tumor Study Group (GSF–GETO) who received 33 treatment lines of targeted therapies, off-label use is already quite common ^79^. Prospective trials will be essential to define their role or that of any other new treatments which may arise.

Unfortunately, osteosarcoma tumor matrix often prevents capturing the effects of investigational treatments by conventional radiologic Response Evaluation Criteria In Solid Tumors (RECIST): lesions simply cannot shrink, even if the tumor cells are killed. Accordingly, a retrospective analysis of seven COG osteosarcoma phase II trials found all drugs inactive on the basis of radiographic response ^80^. Other trial designs and endpoints have been called for ^81^, and COG has constructed baseline EFS outcomes – including 12% EFS at 4 months for patients with measurable recurrent or refractory disease – that could be used as comparators for future phase II trials ^80^. EFS-based outcomes have already been employed in sequential phase II trials of sorafenib given alone ^82^ or in combination with everolimus ^83^ and have demonstrated some activity. Antagonists of the insulin-like growth factor type-1 receptor (IGF1R) are examples of other agents which have shown limited activity in several trials ^84, 85^; however, their development was more or less terminated after they failed in common cancers.

Both sorafenib and IGF1R inhibitors were tested because the focus of osteosarcoma research has shifted towards gaining a better understanding of the driving forces behind tumor development and progression and then hypothesis-driven drug discovery and development ^86^. The identification of the phosphatidylinositol 3-kinase/mammalian target of rapamycin (PI3K/mTOR) pathway as a central vulnerability for therapeutic exploitation and subsequent detection of responsiveness of osteosarcoma cell lines to PI3K/mTOR inhibition ^87, 88^ or the detection of BRCAness in a substantial subset of osteosarcomas ^89^ with the subsequent demonstration of susceptibility of osteosarcoma cells with a BRCAness signature to poly(ADP-ribose) polymerase (PARP) inhibition ^90^ may serve as current examples of preclinical endeavors which deserve clinical evaluation.

It can only be hoped for that not only will we manage to learn more about the biology of osteosarcoma but also this will lead to further steps towards its eradication. Only time, dedicated pre-clinical research, and well-designed clinical trials will tell.

## Abbreviations

ASCT, autologous blood stem cell transplant; CI, confidence interval; CIRT, carbon-ion radiotherapy; COG, Children’s Oncology Group; CNB, core needle biopsy; CT, computed tomography; EFS, event-free survival; EURAMOS, European and American Osteosarcoma Study Group; HDCT, high-dose chemotherapy; HD-MTX, high-dose methotrexate; IGF1R, insulin-like growth factor type-1 receptor; L-MTP-PE, liposomal muramyl tripeptide phosphatidylethanolamine; MAP, methotrexate, Adriamycin, and cisplatin; MAPIE, methotrexate, Adriamycin, cisplatin, ifosfamide, and etoposide; MRI, magnetic resonance imaging; mTOR, mammalian target of rapamycin; PI3K, phosphatidylinositol 3-kinase; PET, positron emission tomography; STIR, short time inversion recovery.
